# The use of a single-piece bone flap for cranial reshaping in anterior craniosynostosis patients: clinical experience and a description of a novel technique

**DOI:** 10.1186/s40902-021-00332-4

**Published:** 2022-01-05

**Authors:** Hatan Mortada, Ikhlas Altuawijri, Taghreed Alhumsi

**Affiliations:** 1 Plastic Surgery Division, Surgery Department, King Saud University Medical City (KSUMC), King Saud University, Riyadh, Saudi Arabia; 2 Department of Plastic Surgery & Burn Unit, King Saud Medical City, Riyadh, Saudi Arabia; 3 Neurosurgery Division, Surgery Department, King Saud University Medical City (KSUMC), King Saud University, Riyadh, Saudi Arabia

**Keywords:** Craniosynostosis, Reconstruction, Bone flap, Cranial reshaping, Plagiocephaly, Trigonocephaly

## Abstract

**Background:**

Craniosynostosis is known as premature closure of one or more of the cranial sutures. Anterior craniosynostosis involves anterior plagiocephaly and trigonocephaly. One of the issues in anterior craniosynostosis skull reshaping is maintaining an aesthetically pleasing forehead curve. Therefore, in this article, we demonstrate our novel technique to use a single-piece bone flap for cranial reshaping of the anterior mold in patients diagnosed with anterior craniosynostosis. A retrospective record review of patients who underwent single piece bone flap cranial reshaping for correction of unicoronal synostosis (UCS) and metopic synostosis (MS) at an Academic Institute in Riyadh, Saudi Arabia, between 2018 and 2020, was conducted.

**Results:**

Six non-syndromic consecutive patients were included. Three of the patients had MS. The mean age at surgery was 11.16 months (range, 6–19 months). The average OR time was 315 min (range, 263–368 min). The average intraoperative blood loss was 225 ml (range, 100–400 ml). All patients had achieved acceptable functional and aesthetic results.

**Conclusion:**

Our novel technique is an innovative and efficient reconstructive technique to simultaneously address MS and UCS and minimize intraoperative bleeding and surgery time. However, more studies with more cases are required.

## Background

Craniosynostosis is known as premature closure of one or more of the cranial sutures, which causes an abnormally shaped head. Craniosynostosis occurs in about 1 in 1800–2500 newborns. Patients with non-syndromic craniosynostosis (NSCS) usually have only one cranial suture involved. However, when two or more cranial sutures are affected and associated features arise, the patients are labeled to have syndromic craniosynostosis (SCS) [1]. Anterior craniosynostosis involves anterior plagiocephaly and trigonocephaly. Plagiocephaly is a universal phrase indicating the unilateral flattening of the anterior or posterior part of the cranium. Anterior plagiocephaly is caused by unicoronal synostosis (UCS). UCS causes an isolated growth defect, compensatory nearby regions expansion, and noticeable fronto-orbital dysmorphic features [2]. On the other hand, trigonocephaly is a term indicating a congenital cranial deformity caused by premature closure and ossification of the metopic suture; metopic synostosis (MS), and the term itself, is used to express a patient with a wedge-shaped skull [3]. Craniosynostosis reconstructive procedures are commonly performed surgeries in plastic craniofacial surgery and pediatric neurosurgery [4]. The surgical restoration of craniosynostosis has a long history since the 1st surgical technique was described in the late nineteenth century [5]. Many different operative techniques for surgical treatment of trigonocephaly and plagiocephaly have been described in the literature ranging from a minimal suturectomy to calvarial bone remodeling and noninvasive procedures to distraction osteogenesis [6–18]. The choice of procedure depends on the severity and type of craniosynostosis deformity. Surgical reconstruction of anterior craniosynostosis is predominantly performed for psychosocial and aesthetic considerations [9]. One of the issues in anterior craniosynostosis skull reshaping is maintaining the new forehead curve. Therefore, in this article, we demonstrate our novel technique to use a single-piece bone flap for cranial reshaping of the forehead in patients diagnosed with anterior craniosynostosis. We also investigated the surgical correction, results, and intraoperative measures in the patients who have been treated with this novel technique at our institute.

## Methods

### Patients selection, study design, and ethical consideration

A retrospective record review was conducted of patients with anterior craniosynostosis who underwent cranial reshaping by the senior author (T.A.) at our institute, between 2018 and 2020. Patients with anterior craniosynostosis who underwent single piece bone flap cranial reshaping to correct UCS and MS were identified. We excluded syndromic patients and those with hydrocephalus or with intracranial abnormalities. Patient demographics, including age, sex, and weight, were extracted from the medical records. In addition, length of hospital stay, pediatric intensive care unit (PICU) stay time, total operative time, anesthetic duration, intraoperative blood transfusion, estimated blood loss volume (ml), and surgical complications (e.g., infection, bleeding, wound healing issues, seizures, need for exploration) were reviewed. Signed consent to use preoperative and postoperative images for publication was obtained from the parents. This study was authorized by the institutional review board (Ref. No. 20/0984/IRB).

### Description of surgical technique

**Fig. 1:**
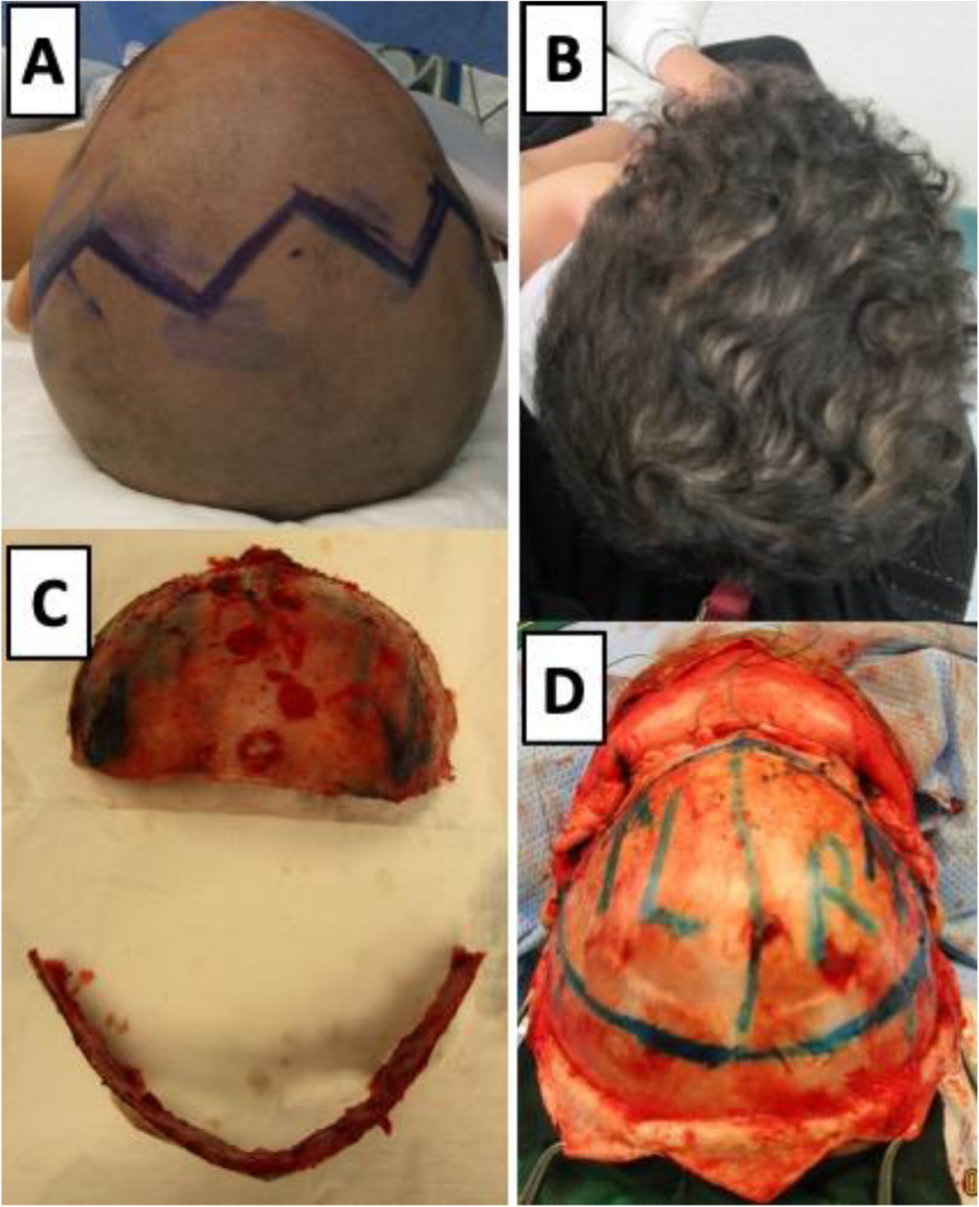
Case of trigonocephaly in a 13-month-old boy with metopic craniosynostosis showing conical shape forehead with ridge. **A** Preoperative images. **B** 9 months after surgery. **C** Forehead and supraorbital bar after remodeling on a side table. **D** Intraoperative images of marking done for cranium removal

**Fig. 2:**
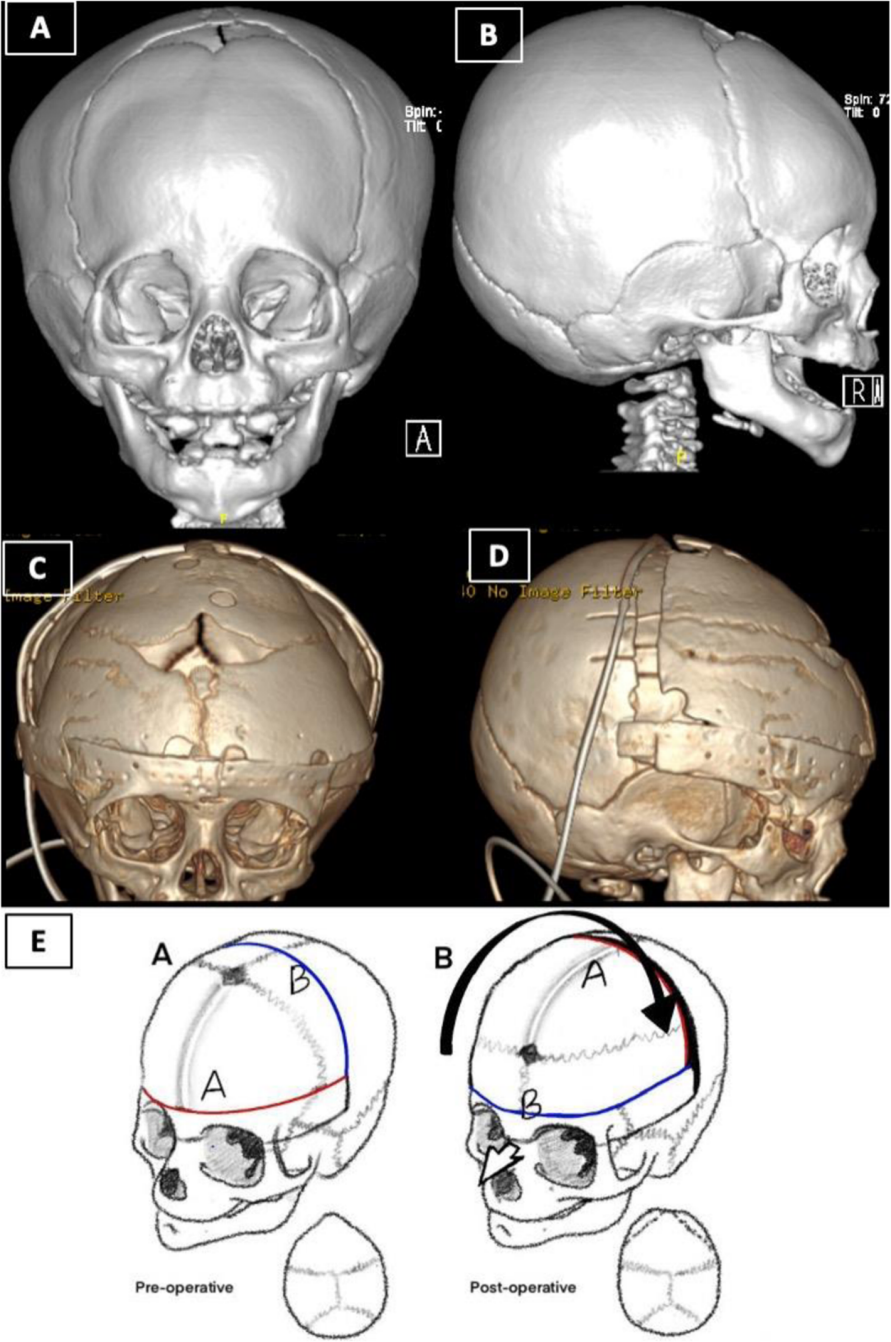
Six-month-old baby girl, case of Trigonocephaly. **A**, **B** Preoperative 3D CT scan. **C**, **D** One-day postoperative. **E** Illustration of the single bone flap that was used in its original position in a trigonocephaly patient as the single bone flap after it was completely removed of anterior vault of the cranium as one piece till supraorbital rim and rotated 180°

**Fig. 3:**
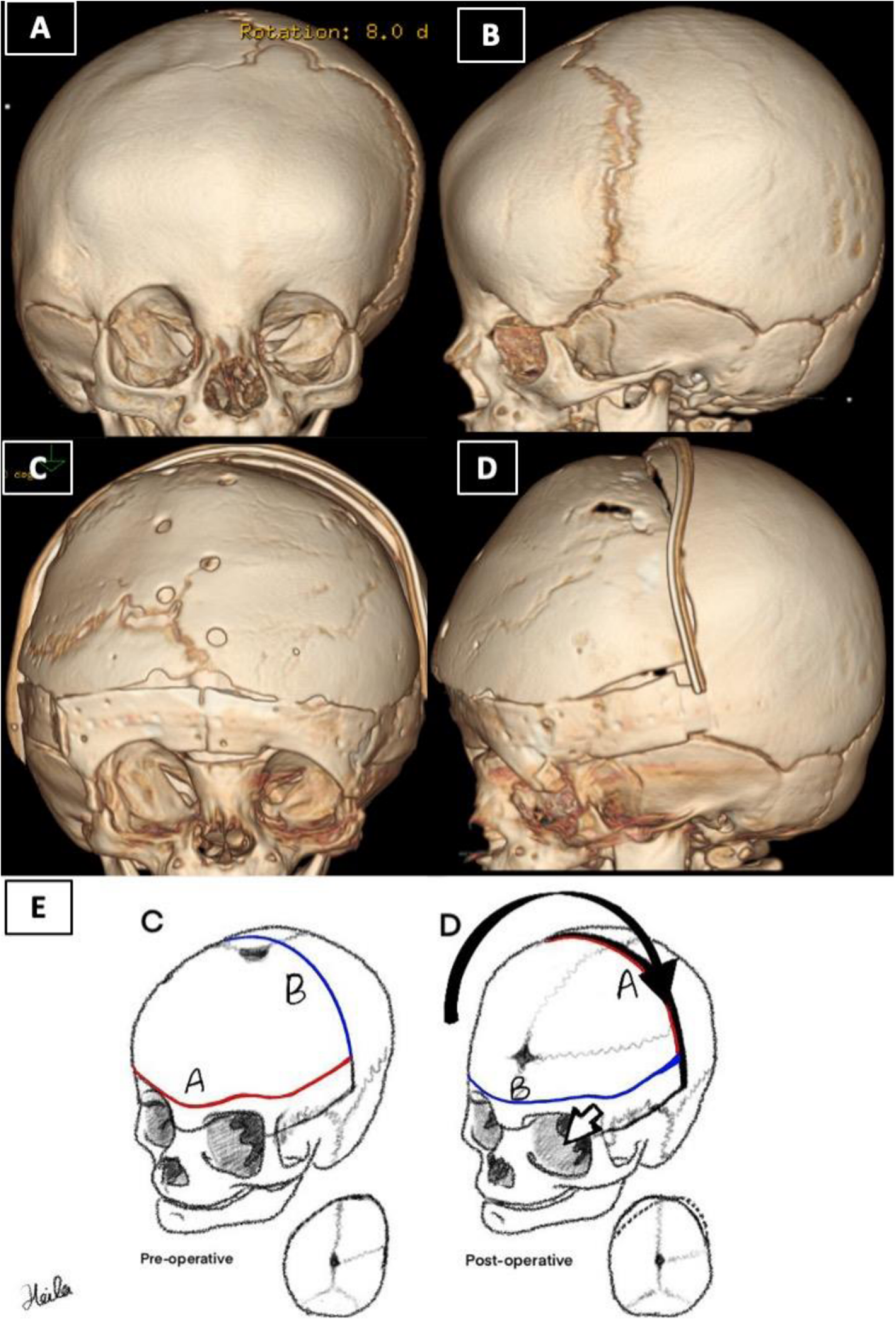
Eight-month-old baby girl, case of plagiocephaly. **A**, **B** Preoperative 3D CT scan. **C**, **D** Day 1 postoperative. **E** The same illustration but in a plagiocephaly patient

Our operative team was composed of a craniofacial plastic surgeon, a pediatric neurosurgeon, and an anesthesiologist. The patient’s diagnosis was based on full history, comprehensive physical examination, neurological examination, and three-dimensional computed tomography scan of facial bones. Complete blood count, creatinine, electrolytes, and blood crossmatch were obtained from all patients preoperatively. All the craniofacial surgeries were performed by the same craniofacial plastic surgeon and pediatric neurosurgeon. The following describes our novel technique’s main elements; however, patient-specific adaptation to each dimension was be made for each unique patient. All patients were operated on under endotracheal intubation and general anesthesia. A warming mattress was used to avoid hypothermia. Prophylactic intravenous antibiotic was given at anesthesia induction and was repeated at due time intraoperatively and postoperatively. Peripheral lines and foley’s catheter were inserted. Patients were positioned in a supine position. The head was shaved and draped in the usual fashion. The bicoronal flap and midline were marked (Fig. 1A). Homeostatic running sutures with a nonabsorbable polypropylene suture were made anterior and posterior to the planned incision line. Tumescent lidocaine and triamcinolone solution (1% lidocaine with epinephrine (weight appropriate dose)+ 40 mg triamcinolone + normal saline 50 cc) was injected subcutaneously. Bicoronal incision in zigzag fashion was carried out from the right ear to the left ear. The bicoronal flaps were elevated, and dissection was performed in the subgaleal plane till 1 cm cephalic to the supraorbital rim. Subperiosteal dissection was then carried out around the supraorbital bar. After that, the craniotomy and supraorbital bar osteotomy marks are marked using methylene blue. Markings of the craniotomy are drawn beyond the deformity site (Fig. 1D). Neurosurgery joins for the craniotomy. Multiple burr holes are created along the line of the sagittal sinus bilaterally and the craniotomy site. The dura is gently separated from the cranium via the multiple burr holes and area of sagittal sinus is carefully dissected through the burr holes. The anterior cranial vault is removed as one single bone piece (Fig. 1C, D). Fronto-orbital bar osteotomy is then performed with meticulous protection of both the globes and brain tissue. Cranial reshaping is then conducted by advancing the bandeau and bone grafts as needed in MS and asymmetric advancement in UCS. The single bone flap is rotated 180°, utilizing the vertex curve to create the new forehead. Resorbable plates, screws, and polydioxanone sutures are used to fix the reconstruction in place (Fig. 2E). The temporalis muscle is resuspended. Hemostasis is secured using cellulose products, absorbable fibrin sealant patch (TachoSil®), bone wax, surgicel snow, and electrocautery throughout the procedure. The closure is then conducted in layers with a medium-sized drain inserted in the posterior flap. The head is then washed and cleaned, and dressing is applied. All outpatients receive intraoperative tranexamic acid transfusion and blood transfusions. All patients in this series were extubated and shifted to the pediatric intensive unit overnight. A three-dimensional CT scan is repeated the next day (Figs. 2A, B and 3A, B). Surgical drains are usually removed 4–5 days post-surgery. All patients are further followed up in the outpatient department to assess their neuropsychologic development and craniofacial growth. Outpatient follow-up is carried out at 3 weeks, 6 weeks, 3 months, 6 months, and 1 year postoperatively (Fig. 1B).

## Results

Six consecutive patients (four females, two males) with anterior craniosynostosis were operated in our institute using this novel technique, Table 1. Three of the patients had MS, whereas three had UCS. All the patients were non-syndromic. The mean age at surgery was 11.16 months (range, 6–19 months), and the mean weight at the time of surgery was 9.55 kg (range, 7.3–12.9 kg). Mean hospital stay was 8.16 days (range, 6–11 days), and all patients stayed 1 day in PICU after surgery. The average OR time was 315 min (range, 263–368 min). The average anesthesia time was 425 min (range, 368–529). The average intraoperative blood loss was 225 ml (range, 100–400 ml), and all patients received an average of 329.166 ml packed red blood cell transfusion (range, 200–700 ml). Only one patient developed a small dural tear intraoperatively. No complications such as intracranial hemorrhage, infection, wound dehiscence, or mortality was encountered in our series. At the time of the most recent clinical evaluation, all patients had achieved acceptable functional and aesthetic results.

**Table 1:** Preoperative diagnosis, number of patients, age at surgery, operating time, and estimated blood loss

Case	Age (months)	Sex	Diagnosis	OT (min)	EBL (ML)	BT (ml)
1	6	Female	MS	290	400	275
2	15	Female	UCS	352	200	700
3	6	Male	MS	368	350	350
4	8	Female	UCS	287	100	250
5	13	Male	MS	263	150	200
6	19	Female	UCS	335	150	200

*OT* operating time, *EBL* estimated blood loss, *BT* blood transfusion

## Discussion

Craniosynostosis is a condition that is primarily managed by surgical intervention. Management aims to offer enough intracranial volume to facilitate brain growth and expansion, to decrease the adverse sequelae on cognitive function, and to create a cosmetically acceptable standard shape of the skull [19, 20]. The history of operative management of craniosynostosis involves “Strip Craniectomies” that was first performed by Lannelounge in 1890 and Lane in 1892. The idea behind strip craniectomies is that minimal release/ removal of the prematurely closed sutures was assumed to allow the head to grow naturally without the need for any significant cranial vault remodeling. Nevertheless, the end outcomes were unpredictable, and the aim of spontaneous normalization and self-correction of the head and fronto-orbital disfigurement was rarely achieved. In 1967, Tessier had implemented the concept of craniotomy for suture release together with skull reshaping using an intracranial and extracranial approach, ingenious osteotomy sites, 360° periorbital dissection, and autogenous bone grafting [21]. The same idea of prematurely closed suture release plus skull reshaping carried out in infancy was carried out by Hoffman and Mohr [22], Whitaker et al. [23], and Marchac and Renier [24]. In the early twentieth century, different surgical techniques were described in the literature to correct anterior craniosynostosis deformity. These procedures range from simple suturectomy to calvarial bone remodeling. Distraction osteogenesis, which is based on Elizavrov’s principle, is also performed [6–18]. Less invasive methods, such as endoscopic suturectomy, have also been mentioned in the literature [25–28]. In our technique, we address the cosmetic outcome in both patients with MS and UCS. The area most noticed by families is the child’s forehead. The authors believe proper attention to the bandeau advancement and symmetry and forehead curvature are key to a successful aesthetic outcome. In our technique, the vertex curve in both these deformities is nicely curved. Utilizing that curve to shape the forehead curve will give a superior aesthetic outcome, along with decreasing operative time and blood loss. We believe that this technique allows for quicker recovery time post-surgery and overall morbidity compared with the standard management

Craniosynostosis surgeries are often performed in the 1st year of life. They are frequently considered complex procedures and may cause significant intraoperative bleeding, ranging from 20 to 500% of the patient’s circulating blood volume [29]. It is well known that blood loss during open repair is significantly higher than other procedures. This may lead to the need for blood transfusion intraoperatively or immediately within the postoperative period. There is limited literature estimating intraoperative blood loss because of the inaccurate measurements of small circulating volumes and the operative logistics. Previously published articles have mentioned the estimated blood loss intraoperatively differs but have documented ranges between 50 and 100% of estimated red cell volume [30–32]. However, these are not objective measurements. Tuncbielk et al. retrospectively reviewed the charts of 30 patients who underwent craniosynostosis repair and reported an average of 566.8 ml blood loss [33]. Another study conducted by Shah et al. reported that the mean estimated intraoperative blood loss was 218 ml in patients undergoing isolated sagittal synostosis repair [34]. A recent study by Lopez et al. showed that the average surgeon estimated blood loss in primary open repair was 207.4 ml [35]. In comparison, our mean estimated intraoperative blood loss was 225 ml (range, 100–400 ml) for all our included patients. We predict that this variance in ranges of estimated blood loss in the literature is highly dependent on the type and complexity of the procedure, age of the patient, and surgeon estimation. Although the open surgical approach for repairing the cranial vault effectively manages craniosynostosis, extensive blood loss, often needing a blood transfusion, has been listed as the most significant risk of undergoing this procedure [30]. According to a 10-year single-center study conducted by Bonfield et al., PRBC transfusion was given to 24% of the patients, 17% were open sagittal, 7% endoscopic assisted sagittal, 6% in unicoronal, 21% bicoronal, 45% metopic, and 45% in patients with multisuture craniosynostosis [36]. Other centers stated that almost 83% of patients who underwent cranioplasty received blood transfusions. This percentage includes all patients (100%) who underwent cranial vault reconstruction, about 98% of patients underwent fronto-orbital advancement and only 32% of spring cranioplasty patients [37]. In comparison, in our study, all patients (100%) received an average of 329.166 ml packed red blood cell transfusion. It is routine to start the blood transfusion at the beginning of the procedure to prevent hemoglobin drop during or after the procedure.

As there are many types of surgical procedures for craniosynostosis, it is expected that operative time will vary with procedure type. Open cranial vault procedure time has been reported to range between 205 and 670 min with a mean of operative time of 342 min [38]. In another retrospective study by Keshavarzi et al., their mean open correction of metopic synostosis surgery was 132.6 min [39]. In our study, the mean operative time was 315 min (range, 263–368 min), which concurs with operative times of open cranial vault remodeling in other centers. In this retrospective study and description of the technique article, we demonstrated our novel technique to use a single-piece bone flap for cranial reshaping of the anterior mold in patients diagnosed with anterior craniosynostosis. As well as, to find the surgical correction results in the patients who have been managed with this new technique at our university hospital compared to similar international papers in comparison to the standard fronto-orbital reshaping for patients diagnosed with anterior craniosynostosis [38, 39], our technique showed better early results and normalization in head morphology. Furthermore, we have revealed a notable decrease in blood loss, recovery time post-surgery, and overall morbidity compared with standard management. The small number of patients may be considered a limiting factor for our series. This might be explained by the lack of early diagnosis or misdiagnosis of the available cases. We believe further studies are needed to modify the technique and assess further surgical outcomes.

## Conclusion

The use of a single-piece bone flap for cranial reshaping of patients diagnosed with anterior craniosynostosis is an innovative, simple, safe, effective, and efficient reconstructive technique to address MS and UCS simultaneously. It can minimize intraoperative bleeding and time of surgery. The technique addresses all the aspects of the deformity and gives superior aesthetic outcomes.

## Data Availability

The datasets used and/or analyzed during the current study are available from the corresponding author on reasonable request.

## Abbreviations

UCS: Unicoronal synostosis
MS: Metopic synostosis
NSCS: Non-syndromic craniosynostosis
SCS: Syndromic craniosynotosis
PICU: Pediatric intensive care unit
OT: Operating time
EBL: Estimated blood loss
BT: Blood transfusion
